# A First Report of Hb Alesha [β67(E11)Val>Met, *G*TG>*A*TG] in an Iranian Patient

**DOI:** 10.29252/ibj.23.6.429

**Published:** 2019-11

**Authors:** Mohammad Hamid, Ebtesam Zargan Nezhad, Hamid Galehdari, Alihossein Alihossein, Gholamreza Shariati, Alireza Sedaghat

**Affiliations:** 1 Department of Molecular Medicine, Biotechnology Research Center, Pasteur Institute of Iran, Tehran, Iran;; 2 Department of Medical Genetics, School of Medicine, Tehran University of Medical Sciences, Tehran, Iran;; 3 Department of Medical Genetic, Faculty of Medicine, Ahvaz Jundishapur University of Medical Sciences, Ahvaz, Iran;; 4 Narges Medical Genetics & PND Laboratory, No. 18, East Mihan Ave., Kianpars, Ahvaz, Iran;; 5 Department of Pediatrics, School of Medicine, Ahvaz Jundishapur University of Medical Sciences, Ahvaz, Iran

**Keywords:** Anemia, Hemoglobin Alesha, Mutation

## Abstract

**Background:**

Hemoglobin (Hb) Alesha is a rare and very unstable Hb variant, resulting in disruption of the heme pocket and producing severe hemolysis in heterozygous statues. In this study, we describe the first report of this variant in an Iranian boy originated from south of Iran with severe hemolytic anemia and mild splenomegaly.

**Methods:**

A six-year-old boy from Khuzestan Province and his parents were studied. Gap-PCR and direct sequencing were performed to detect the -globin gene deletions and β-globin gene mutations, respectively.

**Results:**

The subject had a sporadic mutation GTG to ATG (Val [valine]>Met [methionine]) at codon 67 in heterozygous form on β-globin gene, which was not detected in his parents.

**Conclusion:**

Since both parents proved to be normal, this Hb variant could be considered as a *de novo *mutation, which is highly useful for prenatal diagnosis.

## INTRODUCTION

Most hemoglobin (Hb) variants result from single amino acid substitutions in α- or β- globin structures. Although many of these variants are harmless and not associated with any clinical disease, they may show clinical manifestations that lead to clinical disorders.

Unstable Hb Alesha is caused by a G>A mutation at codon 67 of β-globin gene [β67(E11)Val>Met, *G*TG>*A*TG], changing valine (Val) to methionine (Met) amino acids^[^^1^^-^^3^^]^. This unstable Hb variant was first named Hb Bristol and reported in a 15-year-old Russian boy with severe hemolytic anemia and also in a British patient in which structural study showed that Val was replaced to aspartate (Asp) at codon 67^[^^4^^]^. A complementary experiment using both protein and DNA sequencing of the British patient showed that the primary reported mutation of Hb Bristol known as βV67D that was performed by using protein study was not a correct one; the correct mutation of Hb Bristol was identical to Hb Alesha mutation (β 67[E11] Val to Met). This difference is due to a posttranslational mechanism in which the translated Met converts into an Asp residue^[^^3^^]^. The Met to Asp residue modification is probably done through an oxidative reaction due to the vicinity of the Met side chain to heme iron and the bound O_2_. It has been recommended that 

post-translational conversion of Met to Asp might carry out an unstable molecule via creating a polar residue with negative charge in the middle of the heme pocket causing malformation of the polar bonds between the globin chain and the heme group. This alteration can consequently cause an unstable production of Hb molecule and a severe hemolysis^[^^1^^].^ Therefore, both Hb Bristol and Alesha have the same entity, and the disease is called Hb Bristol-Alesha^[^^3^^]^. In this investigation, for the first time, we pointed out a mutation called Hb Alesha in a six-year-old Iranian boy who suffered from a severe hemolytic anemia and required frequent blood transfusions with mild hepatosplenomegaly.

**Fig. 1:**
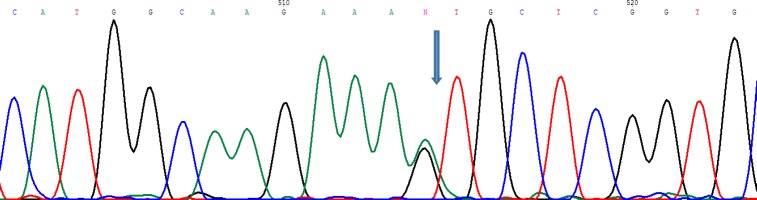
DNA sequencing of β-globin gene showing G>A mutation at codon 67 in heterozygous status.  The arrow shows the position of mutation

## MATERIALS AND METHODS

After obtaining written informed consents, fresh peripheral blood samples from the patient and his parents were collected in EDTA tubes as anticoagulant. The analysis of red blood cell indices and Hb analysis were carried out according to the standard methods. Following the experiment, the molecular studies were conducted on genomic DNA isolated from peripheral blood cells by a salting-out procedure. For investigation of common Mediterranean -globin gene deletions, Gap-PCR was performed as described elsewhere^[^^5^^]^. Sequencing of the β-globin PCR products was conducted by an ABI-3130 Prism Genetic Analyzer (Applied Biosystems, USA).

## RESULTS

In the present study, we represent an Alesha Hb mutation in a six-year-old Iranian boy of Lor ethnicity from Khuzestan Province in Iran as a first case report. Hb Alesha has not been detected by cellulose acetate electrophoresis at alkaline pH (8.4-8.6); so it is considered as an unstable Hb variant. Testing for Hb instability was done by the isopropanol precipitation and heat methods which showed positive results in the subject.

Directed sequencing of β-globin gene of subject and his parents showed that the subject has a novel mutation GTG to ATG (Val>Met) at codon 67 in heterozygous form on β-globin gene but this mutation was not observed in his parents. Therefore, in our subject this Hb variant is probably caused by a kind of *de novo* mutation. The sequencing chromatogram of this mutation is shown in Figure 1. It is important to confirm that target patient had no familial history of anemia and his parents are not consanguineous. The hematological parameters and the molecular features of subject and his parents are shown in Table 1.

## DISCUSSION

The Hb Alesha or Hb Bristol is a rare and very unstable Hb molecule that most patients require frequent blood transfusions and splenectomy. This Hb variant had a wide variety of clinical manifestations, due to introduction of the larger Met residue into the heme pocket, and loss of the bonds between Val at β67 and the heme group^[^^6^^]^.

According to this study and the previous analysis^[^^1^^-^^3^^,^^6^^,^^7^^,^^8^^]^that were carried out on the Hb Alesha-Bristol, it has been confirmed that this mutation is always caused as a result of a *de novo* mutation. It has also been reported in subjects of different origins, three from Japan, two from Russia, as well as one from each of German, Argentina, Brazil, China, and Britain, suggesting that this mutation is not dependent on especial origins^[^^7^^,^^9^^]^. Moreover, the similar mutation has been reported in α-globin chain (α62(E11) Val to Met, i.e. Hb Evans) and γ-chain (γ67(E11) Val>Met, i.e. Hb Toms River). The Hb Toms River is caused by mutation at the conserved γ67 Val residue in fetal Hb that is associated with cyanosis and anemia. Interestingly, biochemical studies have indicated that the Val to metionine substitution at this subunit generates a stable and low oxygen affinity variant of γ-globin, resulting in cyanosis without anemia, but if Met is modified into a Asp molecule which is resulted from post-translational modification produce an unstable variant γ-globin with severe hemolytic anemia. The main reason of the differences in phenotype between the patients with Hb Alesha-Bristol and Hb Toms River is probably due to a conversion rate of Met to Asp^[^^10^^-^^13^^]^. 

**Table 1:** Hemoglobin analysis results and α- and β-globin genes genotypes

**Variable**	**Patient**	**Mother**	**Father**
Age (y)	6	60	75
MCV (fL)	108.6	85.9	88.3
MCH (pg)	34.3	27.7	29.7
RBC (10^2^/L)	2.16	4.33	4.51
Hb (g/dL)	6.5	12.0	13.4
HbA2 (%)	2.1	2.1	2.4
HbF (%)	4.1	0.1	0.1
α-genotype	α^3.7^/αα	α^3.7^/αα	αα/αα
β-genotype	β^Alesha^/norm	Norm/norm	Norm /norm

Norm, normal

We conclude that in patients with hemolytic anemia might not find any mutation in parents of index case because of *de novo *mutation. Therefore, identifying different mutations in affected patient just by indirect (i.e. RFLP linkage) methods is not sufficient and direct mutation detection is also required. As a final point, our result could be highly useful to be considered as an important tool for prenatal diagnosis.
